# Hunger increases delay discounting of food and non-food rewards

**DOI:** 10.3758/s13423-019-01655-0

**Published:** 2019-09-13

**Authors:** Jordan Skrynka, Benjamin T. Vincent

**Affiliations:** Division of Psychology, School of Social Sciences, University of Dundee, Dundee, DD1 4HN UK

**Keywords:** Hunger, Valuation, Delay discounting, Inter-temporal choice

## Abstract

How do our valuation systems change to homeostatically correct undesirable psychological or physiological states, such as those caused by hunger? There is evidence that hunger increases discounting for food rewards, biasing choices towards smaller but sooner food reward over larger but later reward. However, it is not understood how hunger modulates delay discounting for non-food items. We outline and quantitatively evaluate six possible models of how our valuation systems modulate discounting of various commodities in the face of the undesirable state of being hungry. With a repeated-measures design, an experimental hunger manipulation, and quantitative modeling, we find strong evidence that hunger causes large increases in delay discounting for food, with an approximately 25% spillover effect to non-food commodities. The results provide evidence that in the face of hunger, our valuation systems increase discounting for commodities, which cannot achieve a desired state change as well as for those commodities that can. Given that strong delay discounting can cause negative outcomes in many non-food (consumer, investment, medical, or inter-personal) domains, the present findings suggest caution may be necessary when making decisions involving non-food outcomes while hungry.

## Introduction

It is beneficial to have evolved behaviors to homeostatically correct undesirable physiological or psychological states. Increasing the subjective value placed on more immediately available rewards is a plausible feedback mechanism to behaviorally correct an undesirable state. For example, we see that delay discounting of cigarettes increases in a nicotine-deprived state, biasing choice towards smaller sooner nicotine rewards at the expense of larger but later rewards (Field et al., 2006). Similarly, mild opioid deprivation leads to increased discounting of heroin rewards for dependent individuals (Giordano et al., 2002). This is also the case outside of substance dependence—discount rates for food rewards are higher when participants are hungry relative to sated (Button, 2017; Kirk & Logue, 1997), and induced feelings of relative deprivation result in increased discounting for monetary rewards (Callan et al., 2011).

**Fig. 1:**
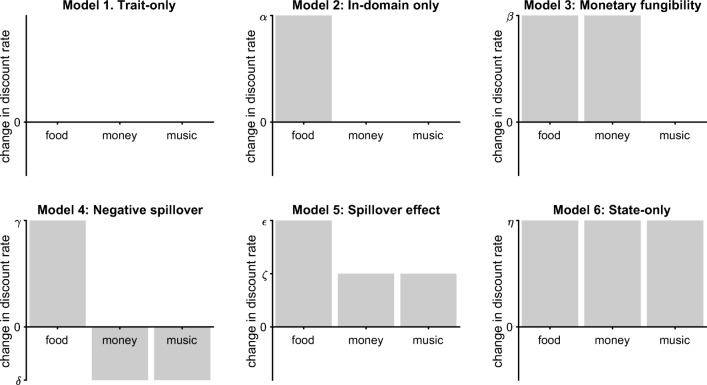
Predictions of the models for a repeated-measures context (control vs. hunger conditions). The *x*-axis shows the commodity where food is the in-domain commodity, and money and music downloads are two out-of-domain commodities. Music downloads were chosen as an out-of-domain reward with no feasible route to affect hunger state (see text). The *y*-axis shows predictions in terms of a change in discount rate (increase in discount rate = increased delay discounting) for an individual going from control to fasted states. *Bars* are schematic only, with changes being determined by model parameters (shown by Greek symbols) to be estimated from the data. These parameters are free to vary within the following constraints: *α* > 0, *β* > 0, *γ* > 0, *δ* < 0, *𝜖* > *ζ* > 0, *η* > 0

But how do our valuations systems alter discounting of out-of-domain commodities whose acquisition would not directly achieve homeostasis? There is evidence that various state manipulations affect general discounting for monetary rewards, such as sexual cues (Van den Bergh & Dewitte, 2008), hunger (Bartholdy et al., 2016; Wang & Dvorak, 2010), and nicotine withdrawal (Field et al., 2006) all result in increased monetary discounting. However, discounting of monetary reward may be a special case; because of its highly fungible nature, our valuation system may increase its discounting of money to indirectly achieve a desired state change through consumer transactions. What is rather less clear is how our valuation systems modulate discounting for the wide range of other out-of-domain commodities with no apparent route to achieve the relevant state change. We can enumerate six candidate models (Fig. 1; ranging from more trait- to more state-based accounts) of how this may work.

1. The *trait-only model* asserts that state changes have no effect on the delay discounting of any commodity.
2. The *in-domain only model* predicts that delay discounting will be altered only for in-domain commodities that can directly cause a desired state change, and discounting for out-of-domain commodities will be unaffected.
3. The *monetary fungibility model* predicts that delay discounting will be increased for the in-domain commodity as well as for money as it can indirectly cause a desired state change.
4. The *negative spillover model* predicts that discounting of the in-domain commodity will be increased, but that discounting of out of domain commodities will be decreased.
5. The *spillover model* predicts that delay discounting will be increased for in-domain commodities, and that there is a smaller spillover increase in delay discounting for out-of-domain commodities.
6. The *state-only model* proposes that state changes will equally affect discounting for all commodities when placed on a comparable scale.

The literature cannot currently differentiate between these, as there is evidence to support all six of these mutually incompatible models of valuation change. A reasonable argument can be made that discounting has *trait-like* properties (see review by Odum 2011). That discount rates are correlated with various personality traits (Mahalingam, Stillwell, Kosinski, Rust, & Kogan, 2014) suggests that discounting also has a trait-like nature. Discount rates have also been shown to be stable over time—high test–retest correlations have been reported with tests conducted 1 week apart (Simpson & Vuchinich, 2000), and even up to 1 year apart (Kirby, 2009). There is also consistency in discounting across multiple reward types—discount rates for food, money, music, films, and books are all positively correlated (Charlton & Fantino, 2008), suggesting an underlying trait-like discounting construct. However, discounting also shows *state-like* properties, reviewed by Odum and Baumann (2015). Discount rates are modulated by context (Dixon et al., 2006), arousal (Lempert et al., 2016; Van den Bergh & Dewitte, 2008), as well as levels of estradiol in naturally cycling women (Lucas & Koff, 2017; Smith et al., 2014). A state-only explanation is consistent with the dual-systems approach of Metcalfe and Mischel (1999). If an undesirable state triggers the putative hot system, then the prediction is that a broad-spectrum increase in discount rates would follow. The results of Li (2008) provide empirical support for this hypothesis—consumers exposed to arousing food pictures or cookie scent had broad-spectrum effects across domains. For example, they were more likely to choose camping over studying, an attractive over a competent job candidate, or a movie ticket over a book token. There is also evidence however that our valuation systems are tuned specifically to commodities that can correct a deviation from a desired state (i.e., the *in-domain* model). Libedinsky et al. (2013) show that sleep deprivation increases discounting of effort but had no effect upon discounting of money, and Mitchell (2004) showed nicotine deprivation increased discounting for immediate cigarettes versus delayed money but not for immediate money versus delayed money. Then again, there is also evidence for a special status for discounting of money because of its fungibility that would support the *monetary fungibility* model. A number of studies have observed an increase in discounting for money, based upon non-monetary state changes such as: hunger (Wang & Dvorak, 2010), emotional arousal (Lempert et al., 2016), sexual arousal (Van den Bergh & Dewitte, 2008), and nicotine deprivation (Field et al., 2006). However, these results can also be seen as consistent with the *spillover* model—it could have been that those state changes had spillover effects to other commodities which simply went unmeasured. For example, men who are placed in a hot affective state (via sexually suggestive stimuli) have a higher discount rate for money (and out-of-domain commodity) than men in a control cold state (Van den Bergh & Dewitte, 2008). This could also be due to money’s role as a status enhancer, or its potential use to purchase sexual gratification (Ariely & Loewenstein, 2006), but the authors argue that state changes in one domain may have effects on discounting in other domains. This could also be seen as a less strong version of the *state-only* model but where discounting for out-of-domain commodities are affected less than in-domain commodities. Finally, there is also reason to believe that we emphasize certain commodities by devaluing others (Brendl et al., 2003) in a kind of *negative spillover effect*, but as far as the authors are aware, this has not been proposed specifically in relation to temporal discounting. The results of (de Ridder et al., 2014) are partially in support of this model. They found that discounting of money decreased when hungry. However, their hunger group participants had (confusingly) lower discounting for food than the control group, so it is difficult to interpret these findings in the context of previous studies. This may have been due to the between-participant design, rather than employing a repeated-measures approach.

Because of this mixed evidence, this study aimed to characterize the precise effect of a deprivation state upon the discounting of both in- and out-of-domain commodities. We chose to focus upon the widely experienced state of hunger, which we induced by a short period of fasting. We took repeated measures of individuals’ discount rates in both control and hungry states. In order to differentiate the six models outlined, we assessed discount rates for multiple commodities. Discounting for food was assessed because of its direct ability to correct the hunger state. We also assessed delay discounting for money, which is processed symbolically not metabolically (Charlton & Fantino, 2008) although it is indirectly capable of correcting the undesirable hunger state through its exchange value. Finally, we assessed delay discounting for song downloads, as there is no clear mechanism for music to influence the hunger state.

## Methods

### Design

A 2 × 3 repeated-measures design was implemented to study the effect of hunger (control vs. hunger conditions) upon the delayed reward preferences of three commodities (food, money, music) for each participant. The hunger condition involved fasting, which we detail below. The participant’s subjective hunger scores were also measured 1–2 weeks apart (*M* = 9 days). The order of control and hunger conditions were randomized for each participant. Participants were not rewarded financially, nor with course credits.

### Participants

Fifty participants (28 females and 22 males with mean age of 21.7) were recruited via university e-mail and social media. We used a repeated-measures design so *N* = 50 for all measures.

Because Bayesian analyses of the data were planned, we did not specify a sample size in advance. Our stopping rule was based upon practical and time constraints—no preliminary data analysis was conducted before data collection finished. Part way through data collection, we visualized raw delay discounting behavior to confirm our adaptive discount rate measure (see below) was working. The sample size is appropriate for the repeated-measures context of the study, and Bayesian analyses provides credible intervals and Bayes factors such that we can avoid unfounded confidence in the study outcomes. With only one measure per person per condition, we had no intention to conduct a participant-level analysis of the data.

No participants reported known issues that may affect their blood glucose levels when fasting. We verbally assessed compliance with the hunger and control state instructions, with zero reported lapses. Only seven participants had fasting blood glucose above typical upper limits of fasting levels, but this level was only marginally exceeded and so we did not remove any participants based on suspected non-compliance (see Supplementary Material for more details).

### Experimental procedure

The study was given ethical approval by the Research Ethics Committee of the University of Dundee before participants were recruited. Participants were informed of the procedure for both sessions before they provided written consent.

In the control condition, participants were asked to eat in the 2 h prior to being tested. In the fasted condition, participants were asked to fast for 10 h prior to being tested. All testing in the fasted condition involved a slightly extended overnight fast as all testing was conducted in the morning. The order of control and hunger conditions was random for each participant. The procedure of each session was: measurement of blood glucose levels, subjective hunger questionnaire completion, then delay discounting was measured with a delay discounting choice task. Verbal debriefs were only given after each participant’s second visit to the lab.

### Subjective hunger measure

The Food Craving Questionnaire-State (FCQ-S) (Cepeda-Benito et al., 2000) was used to measure state-dependent food cravings. Participants responded using a five-point Likert scale on 15 questions, each focusing on one of five characteristics: an intense desire to eat; an anticipation of positive reinforcement that may result from eating; anticipation of relief from negative states and feelings as a result of eating; obsessive preoccupation with food or lack of control over eating; and craving as a psychological state.

### Delay discounting measure

Delay discounting behavior of participants was measured in both control and fasted states using a Bayesian adaptive procedure (Vincent & Rainforth, 2018). There are many good adaptive methods of eliciting inter-temporal preferences but this approach is notable, as it allows us to near-optimally maximize the precision of the estimates of discount rates for a given number of trials.

In each condition (control vs. fasted), three separate delay discounting tasks were conducted for each commodity (money, food, music downloads) in a randomized order. The wording of the questions was adapted to be appropriate for each commodity, for example “*£A* now, or *£B* in *DB* [hours, days]” or “*A* chocolate bars now, or *B* chocolate bars in *DB* [hours, days]”. All participants completed 35 delay discounting trials.

A fixed delayed reward protocol was used; the value of *B* was fixed for each commodity—at 20 for money and music, and ten for food (see below). The adaptive procedure picked the delay *DB* as well as the immediate reward magnitude *A*. The delayed reward could take on 19 possible delays, approximately logarithmically spaced between 1 h and 1 year. A full explanation of the methods and a comparison to other approaches is provided by Vincent and Rainforth (2018).

Participants read an instruction sheet adapted from Odum and Rainaud (2003) that explained the discounting task and stated that they would not receive the reward they chose, but to make decisions as though they were really going to receive the reward.

In order to compare discount rates for each commodity, an exchange rate was set at *£*20 = 20 song downloads = 10 chocolate bars. The largest value of each commodity was equal to *£*20 based on the current average market cost for one unit of each commodity. Inspired by Odum et al., (2006), we used this moderate upper bound on the reward magnitude to avoid presenting participants with unrealistic amounts of delayed food reward.

### Other measures

We also measured participants’ momentary blood glucose concentration in each testing session. We omit presenting the analysis of this data here because of potential methodological limitations, however this data is presented in the Supplementary Material.

### Scoring discount rates

We assume the commonly used hyperbolic discount function (Mazur, 1987), which models present subjective value *V* as a function of a reward magnitude *R* at a delay *D*, *V* = *R* ⋅ 1/(1 + *k* ⋅ *D*). Here, *k* is the discount rate, which has units of days^− 1^. Because *k* is known to be very positively skewed, we expressed our prior beliefs as normally distributed over log(*k*).

We estimated the full posterior distribution over log discount rates given the data, *P*(log(*k*)|data), where the data consisted of the raw trial data from the delay discounting experiment. This was done for each participant × condition combination separately and independently. Data columns were: *A* and *B* the reward values for the immediate and delayed choices, respectively, a delay for the immediate choice *D*^*A*^ = 0 and a delay *D*^*B*^ for the delayed choice and *R* for the participant’s response. The following probabilistic model was used:

$$ \begin{array}{@{}rcl@{}} \alpha &\sim& \text{Exponential}(0.1)\\ \epsilon &\sim& \text{Beta}(1.1,10.9)\\ \log(k) &\sim& \text{Normal}(\log(1/50), 2.5)\\ {V^{A}_{t}}  &=& A_{t} \cdot \frac{1}{1+k\cdot {D^{A}_{t}}}\\ {V^{B}_{t}}  &=& B_{t} \cdot \frac{1}{1+k\cdot {D^{B}_{t}}}\\ P_{t}  &=& \epsilon + (1 - 2 \epsilon) \cdot {\Phi} \left( \frac{{V^{B}_{t}} - {V^{A}_{t}}}{\alpha} \right)\\ R_{t}  &\sim& \text{Bernoulli}(P_{t}) \end{array} $$

where *t* is a trial, corresponding to a row in the raw data table, and Φ(⋅) is the cumulative normal distribution. In practice, *P*(log(*k*)|data) was computed using Markov chain Monte Carlo methods described by Vincent (2016).

Because our Bayesian parameter estimation procedure can produce model predictions for the probability of choosing the delayed reward on each trial (*P*_*t*_), we are able to assess the ability of the discount function to account for behavioral data using signal detection theory (Wickens, 2002). The area under the receiver operating characteristic curve gives the model’s ability to correctly predict the inter-temporal choice data—a value of 1 means perfect prediction, 0.5 means chance level, and between 0.5 and 0 means below chance level.

### Model comparison

We used computer modeling of changes in discount rates for each of the three commodities, to quantitatively evaluate the plausibility of the data under each of the four models. Changes in log discount rate Δ_*p*, *c*_ for participant *p* and commodity *c* were modeled as Cauchy distributed around the group mean with a certain variance (termed scale for the Cauchy distribution). Each model consisted of four parameters (although some of these are fixed at zero) corresponding to group-level change in log(*k*) for food, money, and music, and the scale of participant-level deviation from group changes. The parameters of each model were: *𝜃*_1_ = {*α*,0,0,scale_1_} (2 free parameters); *𝜃*_2_ = {*β*, *β*,0,scale_2_} (2 free parameters); *𝜃*_3_ = {*γ*,−*δ*,−*δ*,scale_3_} (3 free parameters); *𝜃*_4_ = {*𝜖*, *ζ*, *ζ*,scale_4_} (3 free parameters). All parameters were constrained to be greater than zero. For each model, we calculated the best fitting (maximum likelihood) parameters (*𝜃*_*m*_) using the likelihood function below which sums the log probabilities of the data Δ_*p*, *c*_ for given parameter values.

$$ \begin{array}{@{}rcl@{}} P(\mathrm{data|\theta_{m}}) &=& {\Sigma}_{p=1}^{P} {\Sigma}_{c=1}^{C} \log(\text{Cauchy}({\Delta}_{p,c}; \\ &&\text{location} = \theta_{m,c}, \text{scale} = \theta_{m,4} ) ) \end{array} $$

### Data and code availability

The data collected for this study as well as the analysis scripts are available at the Open Science Foundation, https://osf.io/a37wy/. Analyses were conducted in Python and JASP (JASP Team, 2018). We used PyMC3 (Salvatier et al., 2016) to conduct Bayesian parameter estimation for the discount functions, using methods described by Vincent (2016).

## Results

Analysis of the subjective hunger scores showed that the fasting manipulation successfully increased hunger. FCQ-S scores increased by an average of 1.5 points, with a very large paired Cohen’s *d* effect size of 1.8, CI_95*%*_[1.3, 2.3].

We confirmed that the hyperbolic discount function was a good fit to the data. The median of the area under the receiver operating characteristic curve (see Methods) was 0.90 with 95% highest density interval [0.57, 1]. This means the hyperbolic discount function was a very good predictor of the participant’s choice data, and that the estimated discount rates meaningfully capture participant’s delay discounting behavior.

**Fig. 2:**
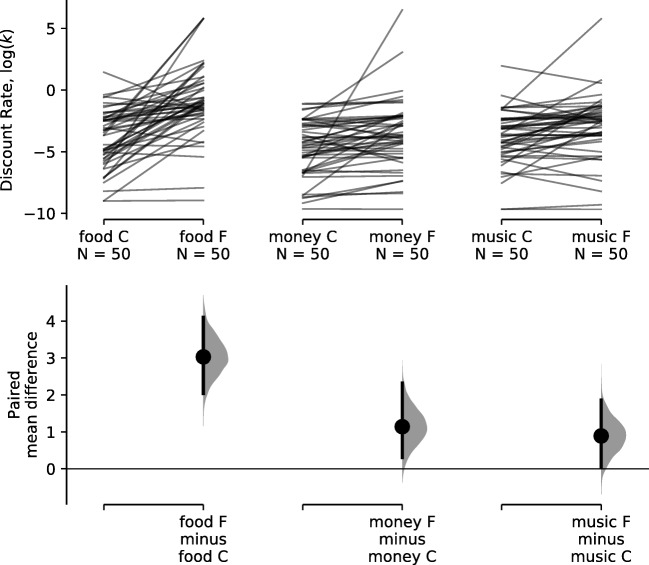
Discount rates for different commodities and conditions. The *top panel* shows the distribution of log discount rates for each commodity and condition combination. The *bottom panel* shows the distribution in participant change in discount rates from control to fasted conditions. *Black points* and *error bars* show the paired mean change in discount rates and 95% confidence intervals, calculated using bootstrap resampling (Ho et al., 2019). The letters “C” and “F” correspond to the control and fasting conditions, respectively. See Supplementary Materials for summary statistics

How did hunger influence discounting behavior? Referring to Fig. 2, average discounting was low for all commodities in the control condition and increased in the fasted condition (see summary statistics in the Supplementary Material). The increase in discounting for food is notably higher than for money and music. This is readily apparent in the lower panel showing the paired mean differences.

The effect of hunger on discounting behavior for food was substantial. The median half life (Yoon and Higgins, 2008) plummeted from 35 days to 3 days in the fasted condition^1^. The effects of hunger on both money and song downloads were similar; the change was less extreme than for money, but still significant. Half life dropped from 90.30 to 40.20 days for money, and from 39.70 to 12.40 days for music. Empirically, the spillover effect (average across participants) for non-food commodities is 24.80% of the effect for food, when measured in log discount rates.

Bayesian repeated measures *t* tests (one-sided) were conducted on the log(*k*) measures to evaluate the effect sizes. This showed a very large (Cohen’s *d*) effect size of 1.30 CI_95*%*_[0.86, 1.75] for food, a medium–large effect size of 0.63 CI_95*%*_[0.24, 1.04] for money and a medium–large effect size of 0.62 CI_95*%*_[0.22, 1.02] for music. While the data were not sufficient to be highly precise about the effect sizes for change in discount rates for money and music, the evidence is decisive for the presence of an increase in discount rates for food (*BF* = 1.94 × 10^6^) and very strong for money (*BF* = 44.6) and music (*BF* = 43.2).

We can assess which of the models (Fig. 1) best accounts for the observed changes in discount rates (Fig. 2, lower panel). Visually, the data seem most consistent with the Spillover model (three parameters), but also potentially consistent with the in-domain model (two parameters). To assess this quantitatively, we calculated Akaike Information Criterion (*AIC*) and Bayesian Information Criterion (*BIC*) values for each of the models as these measures incorporate model goodness of fit, penalize more complex models, and avoid use of priors over parameters for which we have little foreknowledge of in this case (Burnham & Anderson, 2004; Wagenmakers & Farrell, 2004). This results in single *AIC* and *BIC* scores for each model. It is only the relative differences between the scores that are of interest, so we calculate Δ*AIC* and Δ*BIC* by subtracting the *AIC* and *BIC* (respectively) of the lowest (best) score. For both metrics, the Spillover model is the best account of the data (see Table 1). Using the scale of Burnham and Anderson (2004), we find essentially no support for the remaining hypotheses. To get a more intuitive measure, we can convert both *AIC* and *BIC* scores into model weights *w*(*AIC*) and *w*(*BIC*). The Information Criterion scores for model *m* can be converted to model weights by − 0.5Δ*IC*_*m*_ Σ_*i*_(− 0.5Δ*IC*_*i*_), where the denominator is the sum over all models (Burnham & Anderson, 2004). For the Spillover model, we get *w*(*AIC*) = 99.62%, which can be seen as a very high weight of evidence for this model. The Spillover model also results in *w*(*BIC*) = 99.14%, which can be thought of as the posterior probability of this model being true. Overall, we find very strong quantitative support for the Spillover model.

**Table 1:** Formal model comparison results

Model	*n*	LL	Δ(*AIC*)	*w*(*AIC*)	Δ(*BIC*)	*w*(*BIC*)
1. Trait only	1	− 365.66	47.00	0.00	43.18	0.00
2. In-domain	2	− 347.37	12.41	0.00	10.50	0.01
3. Monetary fungibility	2	− 349.16	15.99	0.00	14.08	0.00
4. Negative spillover	3	− 347.37	14.41	0.00	14.41	0.00
5. Spillover	3	− 340.17	0.00	1.00	0.00	0.99
6. State-only	2	− 348.44	14.56	0.00	12.65	0.00

LL is the log likelihood of the data given the maximum likelihood parameters. Higher (i.e., smaller negative) values indicate better fits to the data. Δ*AIC* is the *AIC* value, relative to the best (lowest) *AIC* value, similarly for Δ*BIC*. *w*(*AIC*) and *w*(*BIC*) are the probabilities of each model being the best. Each model has *n* parameters, and all models have a scale parameter for the Cauchy-distributed measurement error of change in log discount rates

To test the robustness of these results, we conducted similar analyses (see Supplementary Material) using the exponential discount function (Samuelson, 1937), the Myerson and Green (1995) hyperboloid, and the modified-Rachlin hyperboloid discount function (Vincent & Stewart, 2019). To compare changes in discounting across different discount functions, we conducted the analyses using the area under the curve metric (Myerson et al., 2001). Using this measure, we found the same pattern of changes in discounting from control to fasting for each of the commodities as that seen in Fig. 2. The same AIC and BIC model comparison procedure resulted in remarkably similar findings for each of the discount functions—the Spillover hypothesis was the best account of the data and there was essentially no support for the remaining hypotheses. In short, we have very strong quantitative evidence for the Spillover model regardless of the precise discount function used to capture participant’s delay discounting behavior.

## Discussion

It is well established that hunger can affect behavior through a wide range of processes, including: food preferences (Lozano et al., 1999), general goal-oriented focus to food (Russell, 2008), social decision making (Aarøe & Petersen, 2013; Strang, 2017), risk preferences (Rad & Ginges, 2017), subjective time perception (Fung, Murawski, & Bode, 2017), behaviors in virtual foraging tasks (Korn, 2015), as well as temporal discounting. For the latter, it has been shown before that a hunger manipulation causes increased discounting for money (Bartholdy et al., 2016; Button, 2017; Wang & Dvorak, 2010) and food (Button, 2017; Kirk & Logue, 1997). The current study additionally shows: (a) hunger brought about by modest fasting periods leads to a *substantial* increase in delay discounting for food; and (b) that there is a roughly 25% spillover to the non-food domain.

In the context of food deprivation, we found very strong quantitative support for the Spillover model capturing how our valuation systems respond, and this is consistent with at least two previous studies. Recently, Otterbring (2019) also found that hunger changes time orientation to focus upon present pleasures and to biases choice towards hedonic rather than healthy food choices. Xu et al., (2015) found that hunger promotes acquisition of non-food objects (such as widescreen TV’s, spa visits, and video cameras) as well as food. However, at this point, we cannot directly assess the external validity of these findings to real-world decision contexts—it may be necessary to explore delay discounting paradigms where participants directly experience rewards and/or the delay waiting period. Although studies comparing hypothetical versus real rewards suggest that this may be a minor concern (Johnson and Bickel, 2002; Madden et al., 2003).

While the Spillover model may indeed be relevant for other contexts, it is too early to be conclusive. If we look at the case of nicotine, while it is established that discounting for money is higher in current smokers than ex smokers than never smokers (Odum et al., 1999), nicotine-deprivation studies would seem to support the in-domain model even though it is similar to food in being a primary reinforcer. A number of studies now suggest that nicotine deprivation selectively increase discounting for cigarettes, but not for monetary rewards (Field et al., 2006; Mitchell, 2004; Roewer et al., 2015). In contrast, mild opioid deprivation leads to increased discounting for both opioids and money (Giordano et al., 2002), more consistent with the Monetary Fungibility or Spillover models. One important factor in arbitrating between the six different models presented in the Introduction, will be to conduct more cross-commodity discounting experiments. It is important to go beyond the primary reinforcer relevant for the particular context (hunger, thirst, drug deprivation) and money, but to also test for other unrelated hedonic rewards to distinguish the Monetary Fungibility and Spillover models.

It will be important to resolve the etiology of this Spillover effect. One view is that it is the result of neural mechanisms which simply do not allow for fine-grained commodity-level valuation changes (Van den Bergh & Dewitte, 2008). Although exploring adaptively rational explanations about *why* this behavior occurs may be a more fruitful approach (Anderson, 1991). For example, it could be that the Spillover effect is part of an adaptation which shifts an agent from exploring their environment to exploiting it, in a reinforcement learning sense (Hills et al., 2015). This was shown by Katz and Naug (2015) who found that hunger shifted honeybee’s behavior towards exploitation of known food resources and away from exploration of potentially larger but unknown future food resources. This is consistent with Xu et al. (2015) who showed that hunger activates a generalized acquisition goal. Alternatively, perhaps the Spillover effect is part of a ‘hedonic homeostasis’ process, where acquisition of out-of-domain commodities may ameliorate some of the negative aspects of the primary in-domain deviation (e.g., hunger, Carver 2015). This is consistent with the recent claim of Otterbring (2019) that discounting is the mechanism which increases hedonic food and non-food choices when hungry, but more work is needed to establish if spillover effects serve an adaptive homeostatic function.

In summary, while it was predictable that delay discounting for food shifts toward immediate gratification when fasted, the evidence shows delay discounting increases even for non-food items. It is certainly not always maladaptive to value smaller but sooner rewards, and so elevated discount rates are not necessarily a bad thing to be avoided. However, in a decision environment where negative long-term outcomes will result from over-valuing the short-term (e.g., consumer, investment, relationship and health-related contexts), the results of this study suggest that decision-makers should be cautious when making decisions while hungry.

## Footnotes

1 Half life (equal to 1/*k*) is the time for a future reward to have half its value, or equivalently, the delay at which you would be indifferent between a reward now or double that reward in the future.
